# CCR2 Regulates Referred Somatic Hyperalgesia by Mediating T-Type Ca^2+^ Channel Currents of Small-Diameter DRG Neurons in Gastric Ulcer Mice

**DOI:** 10.3390/brainsci15030255

**Published:** 2025-02-27

**Authors:** Ziyan Yuan, Huanhuan Liu, Zhijun Diao, Wei Yuan, Yuwei Wu, Simeng Xue, Xinyan Gao, Haifa Qiao

**Affiliations:** 1 Shaanxi Key Laboratory of Integrative Acupuncture and Medicine, Shaanxi University of Chinese Medicine, Xianyang 712046, China; 222040022741@email.sntcm.edu.cn (Z.Y.); 221051212560@email.sntcm.edu.cn (H.L.); diaozhijun@sntcm.edu.cn (Z.D.); 1351023@sntcm.edu.cn (S.X.); 2 Key Laboratory of Acupuncture and Neurobiology, Shaanxi Administration of Traditional Chinese Medicine, Xianyang 712046, China; 3 College of Acupuncture-Moxibustion and Tuina, Shaanxi University of Chinese Medicine, Xianyang 712046, China; yuanwei@sntcm.edu.cn (W.Y.); 2061050@sntcm.edu.cn (Y.W.); 4 Institute of Acupuncture and Moxibustion, China Academy of Chinese Medical Sciences, Beijing 100700, China; 5 Shaanxi Collaborative Innovation Center of TCM Technologies and Devices, Shaanxi University of Chinese Medicine, Xianyang 712046, China

**Keywords:** referred somatic hyperalgesia, dorsal root ganglion, T-type Ca^2+^ channels, the C-C motif chemokine receptor 2, Gastric ulcer

## Abstract

**Background**: Referred pain frequently co-exists with visceral pain. However, the exact mechanism governing referred somatic hyperalgesia remains elusive. **Methods**: By injecting 20% acetic acid into the stomach, we established a mouse model of gastric ulcer (GU). Hematoxylin and eosin (H&E) staining was used as the evaluation criterion for the gastric ulcer model. Evan’s blue (EB) and von Frey tests detected the somatic sensitized area. The DRG neurons distributed among the spinal segments of the sensitized area were prepared for biochemical and electrophysiological experiments. The CCR2 antagonist was intraperitoneally (i.p.) injected into GU mice to test the effect of blocking CCR2 on somatic neurogenic inflammation. **Results**: GU not only instigated neurogenic plasma extravasation and referred somatic allodynia in the upper back regions spanning the T9 to T11 segments but also augmented the co-expression of T-type Ca^2+^ channels and CCR2 and led to the gating properties of T-type Ca^2+^ channel alteration in T9–T11 small-diameter DRG neurons. Moreover, the administration of the CCR2 antagonist inhibited the T-type Ca^2+^ channel activation, consequently mitigating neurogenic inflammation and referred somatic hyperalgesia. The application of the CCR2 agonist to normal T9–T11 small-diameter DRG neurons simulates the changes in the gating properties of T-type Ca^2+^ channel that occur in the GU group. **Conclusions**: Therefore, these findings indicate that CCR2 may function as a critical regulator in the generation of neurogenic inflammation and mechanical allodynia by modulating the gating properties of the T-type Ca^2+^ channels.

## 1. Introduction

Visceral pain is uniquely characteristic because it is perceived in areas of the body that are distant from the affected organs, which is termed referred pain. Referred pain frequently co-exists with visceral pain, as seen in examples such as abdomen pain due to irritable bowel syndrome, pelvis pain due to reproductive problems, or the forelimbs and upper back referred pain caused by coronary heart disease [1,2]. This viscerosensory reflex pathway commences with the visceral primary sensory afferent fiber conducting sensory impulses from the viscera to the central nervous system (CNS), ultimately generating somatically referred pain and peripheral sensitization through the mechanisms of the dorsal root reflex (DRR) [3] and axon reflex (AR) [4]. However, the peripheral neurophysiological mechanisms underlying the intricacies of visceral referred pain and sensitization remain largely unclear.

The cell bodies of visceral afferents reside in the dorsal root ganglion (DRG), characterized as pseudo-unipolar neurons, possessing both central and peripheral axonal processes [2]. This distinctive structure allows both the soma and axon to exhibit electrical excitability, thereby facilitating the continuous transmission of sensory information [5]. Therefore, the DRG is essential in transmitting visceral pain signals to the CNS and peripheral nervous system (PNS). Many studies have shown that DRG neurons are implicated in the development of chronic pain, marked by increased excitability and spontaneous ectopic firing [5,6,7,8]. Nevertheless, the ion channel mechanisms and molecular pathways underlying nociceptor hyperexcitability associated with pain remain incompletely elucidated.

Voltage-dependent Ca^2+^ channels (VDCCs) are pivotal to modulating the excitability of sensory neurons under both normal and pathological conditions [9]. These channels can be categorized into high-voltage-activated (HVA, N, P/Q, L, R-types) and low-voltage-activated (LVA, T-type) subtypes [10]. T-type channels distinctively modulate neuronal excitability through their low-voltage activation and window current properties [11], with functional alterations directly impacting pain thresholds. The activation of the T-type channels enhances the excitability of DRG neurons, contributing to mechanical and thermal hypersensitivity [12]. Conversely, the selective *in vivo* blockade of T-type channels mitigates thermal hyperalgesia and allodynia in rats [13]. Nevertheless, how T-type channels are affected by visceral pain disorders, leading to peripheral sensitization, remains inadequately understood.

T-type Ca^2+^ channels are regulated by different second messenger pathways that respond to the activation of various classes of G protein-coupled receptors (GPCRs) [14]. Several studies suggest that the upregulation of the C-C chemokine receptor 2 (CCR2) expression enhances the excitability of DRG neurons in visceral pain models by sensitizing primary afferent neurons [15]. Additionally, the C-C motif chemokine ligand 2 (CCL2) interacts directly with its receptor CCR2, resulting in increased neuronal firing, which ultimately amplifies nociceptive responses [16,17,18], whereas the CCR2 receptor antagonists partially inhibit T-type channels and elicit analgesia effects [19]. However, it is still not well understood whether the expression of CCR2 on DRG neurons contributes to peripheral sensitization and somatic referred pain hypersensitivity in visceral pain disorders by modulating T-type channels.

In this study, to address these questions, we first established a visceral pain mouse model of gastric ulcers (GU) [20] and identified the referred somatic regions in these mice. Then, behavioral tests were employed to assess somatic hypersensitivity in referred areas of GU mice. Additionally, whole-cell patch-clamping recordings were applied to evaluate the changes in gating properties of T-type Ca^2+^ channels in DRG neurons distributed among the spinal segments of the sensitized regions. With this framework, we investigated the relationship between the role of T-type Ca^2+^ channels and the expression of CCR2 on DRG neurons in GU mice. Finally, we utilized pharmacological methods to evaluate the sufficiency and necessity of T-type Ca^2+^ channels and CCR2 in somatic sensitization and referred pain in GU mice.

## 2. Materials and Methods

### 2.1. Gastric Ulcer Mouse Model

**Figure 1:**
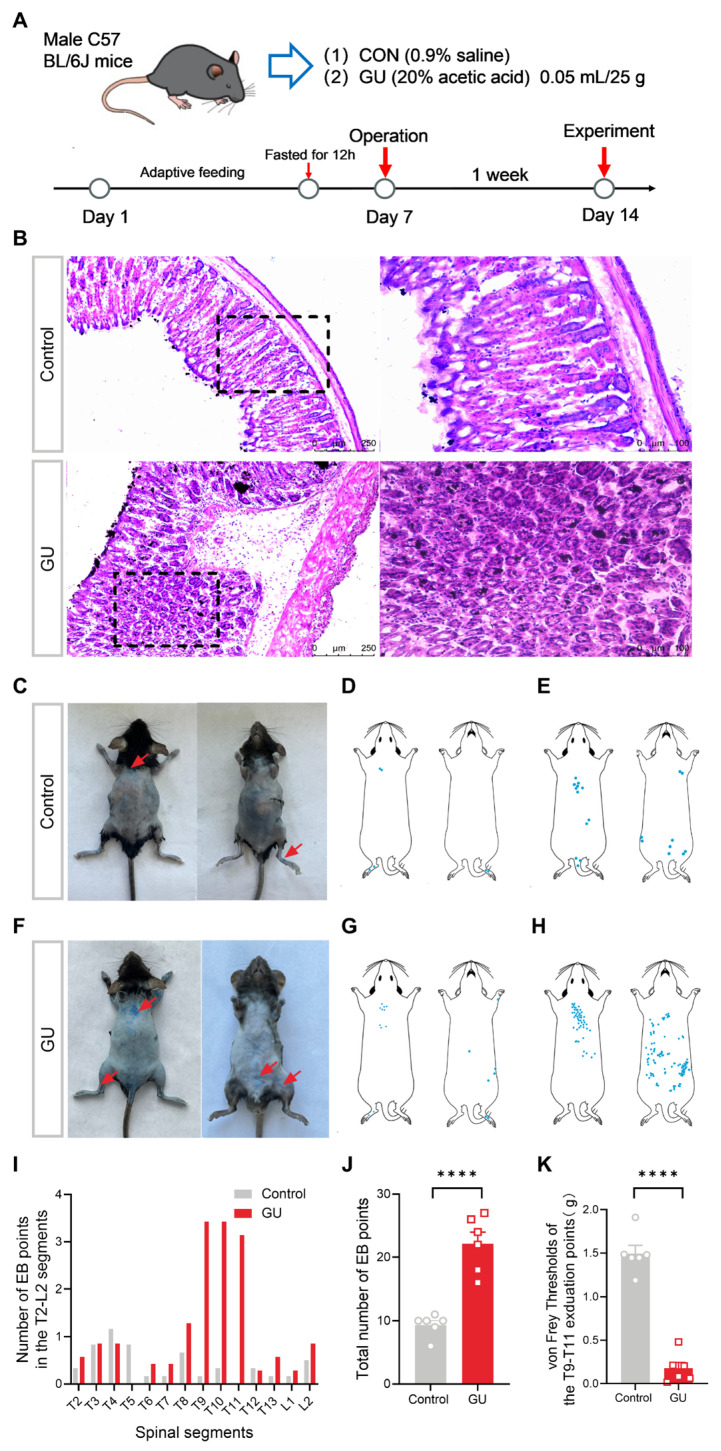
Pathological changes in the gastric ulcer (GU) mice induced by acetic acid. (**A**) Experimental process diagram. (**B**) Representative images of H&E staining of the stomach in control and GU mice. The right panels provide higher-magnification pictures of the boxed regions in the left panels [left: 10× (magnified), scale bar, 250 μm; right: 20× (magnified), scale bar, 100 μm] (n = 6 mice per group). (**C**,**F**) Representative images of EB plasma extravasation points scattered in the regions of operative incision and upper back following GU, as compared to the control. (**D**,**G**) Schematic representation of EB points in the skin from control and GU mice. (**E**,**H**) Schematics of merged EB points in the skin from the control and GU group. (**I**) The number of EB points in the dermatomes of the T2-L2 spinal segments. (**J**) Quantification of total EB points in two groups (n = 6 mice per group). (**K**) Withdrawal threshold to mechanical stimulation in the T9–T11 upper-back exudation points of each group (n = 6 mice per group). The red arrows indicate the locations of EB points, and the blue dots represent the EB points. Compared with the control group, **** *p* < 0.0001.

Male C57 BL/6J mice, weighing between 20 and 25 g, were purchased from Beijing Vital River Laboratory Animal Technology Co., Ltd. (license number: SCXK-2021-0006, Beijing, China). All animal procedures received approval from the Animal Care Committee of the Shaanxi University of Chinese Medicine (Approval ID: SUCMDL20200420003) and followed the National Institutes of Health (NIH) Guide for the Care and Use of Laboratory Animals. The mice were allocated into control (CON) and gastric ulcer (GU) groups for the experiments, as shown in Figure 1A. Ulcers were induced through the administration of acetic acid, following methodologies outlined in previous studies. Under anesthesia, a midline epigastric laparotomy was performed to expose the stomach, into which 50 µL of 20% acetic acid (A6283, Sigma, St. Louis, MO, USA) was injected with a syringe into the sub-serosal layer of the glandular (0.5 mL) [19]. The control group received an equivalent volume of normal saline. Ulcer induction/sham operations were performed by separate investigators. Other experiments were conducted with rigorous randomization and blinded approaches, whereby the experimenter did not know the specific experimental group throughout the procedure.

### 2.2. Histopathological Examination

One week post operation, the mice were anesthetized and perfused with 0.9% saline injection. Then, the gastric tissues were harvested and immersed in 4% paraformaldehyde (PFA) overnight. The gastric tissues underwent dehydration in a gradient concentration sucrose solution and embedded in OCT. Hematoxylin and eosin (H&E) staining was carried out on frozen sections (thickness 14 μm) using an H&E staining Kit (TOP 0446, Biotopped, Beijing, China). The stained sections were examined under a DM2500 digital light microscope (Leica, Wetzlar, Germany) to assess morphology and signs of inflammation.

### 2.3. Detection of Sensitized Points

One week post operation, the mice were subjected to deep anesthetization using 2% isoflurane (R510-22-10, RWD, Shenzhen, China) and were depilated. An insulin needle was employed to inject Evan’s blue (EB) dye (50 mg/kg, 100 μL) into the tail vein. Two hours later, the EB exudation was counted, photographed, and recorded on a diagram illustrating the mouse’s somite locations. The number of EB points in the T2-L2 segment as well as the total number of EB points were, respectively, counted in the control and GU groups.

### 2.4. Behavioral Tests

To determine the referred pain occurred in GU mice, we applied a series of von Frey filaments (ranging from 0.04 to 2.0 g) according to the up–down method previously described [21] to record the mechanical pain threshold on the concentrated blue exudation points of the upper back in mice. Each filament was tested five times for durations of 1 to 2 s. If the mice exhibited a significant escape response, the next lower force filament was applied until two filaments were tested without a positive response. The pressure value of the lowest filament was recorded as the threshold for mechanical pain.

### 2.5. Preparation of Intact Whole-Mount DRG

The mice were intraperitoneally (i.p.) injected with 25% urethane (51-79-6, Sigma, USA) for anesthetization. The intact whole-mount T9–T11 DRG was isolated from mice according to previously established methodologies [6,22]. In brief, the DRG were bilaterally excised and subjected to enzymatic digestion utilizing a mixture of collagenase (2 mg/mL, 9002-07-7, Sigma, USA) and trypsin (1 mg/mL, 10103586001, Sigma, USA) at 37 °C for 15 min, then incubated in oxygenated artificial cerebrospinal fluid (ACSF) for at least 1 h before recording. The recording chamber was saturated with oxygenated ACSF containing (in mM) the following: 140 NaCl, 5 KCl, 10 Glucose, 10 HEPES, 1 MgCl_2_, and 2 CaCl_2_; in addition, pH was adjusted to 7.4 with NaOH.

### 2.6. Electrophysiological Recordings

Whole-cell patch-clamp recordings were conducted at ambient temperature using a Digidata 1550B acquisition system and Axopatch 700B amplifier (Molecular Devices, San Jose, CA, USA) with pClamp 10.7 software. The whole-mount DRGs were visually identified with an upright microscope (BX51WI, Olympus, Tokyo, Japan) equipped with infrared differential interference contrast optics (IR-DIC). Series resistance was compensated by at least 75%, and current traces were corrected using online P/4 trace subtraction. The pipette solution contained (in mM): 130 CsCl, 2.5 MgCl_2_, 10 HEPES, 5 EGTA, 3 Na_2_-ATP, and 0.5 Na_2_-GTP; in addition, pH was adjusted to 7.4 with CsOH. To record Ca^2+^ currents, 1 μM of TTX, 5 mM of TEA-Cl, and 2 mM of 4-AP were added to the ACSF to inhibit sodium and potassium currents.

We applied 0.2 μM of ω-conotoxin MVIIC (N- and P/Q-type channel blocker) and 5 μM of nifedipine (L-type channel blocker) to the ACSF, and then used a 40 ms depolarizing step pulse to record currents from −110 to −40 mV in order to isolate T-type Ca^2+^ currents [12]. Activation curves were evoked with voltage steps from −110 mV to test potentials between −80 and 0 mV in 10 mV increments. Inactivation curves were recorded using a 40 ms test pulse to −40 mV after the 1 s conditioning pulses ranging from −120 to −30 mV with 10 mV increments. Current amplitudes were measured from the peak to the end of the depolarizing pulse. Boltzmann distributions characterized the voltage dependence of activation and steady-state inactivation:

Activation: G/G_max_ = 1/{1 + exp [(V_1/2_ − V_m_)/*k*]}^−1^

Inactivation: I/I_max_ = 1/{1 + exp [(V_m_ −V_1/2_)/*k*]}^−1^

In these formulas, G_max_ denotes the maximal conductance, I_max_ indicates the maximal current amplitude, V_1/2_ is the voltage for half-activated or inactivated, and *k* signifies the slope of the distribution.

### 2.7. Immunofluorescence Labeling

One week post modeling, the mice were anesthetized and underwent cardiac perfusion with saline followed by 4% PFA. The DRG tissues from the T9–T11 segments of the spinal cord were harvested and post-fixed in 4% PFA at 4 °C overnight before being dehydrated in a 30% sucrose solution. Sixteen-micron DRG slices were blocked in phosphate-buffered saline (PBS) with 5% normal donkey serum and 0.5% Triton X-100 for 2 h at room temperature. The sections were then treated with primary antibodies, including mouse anti-Ca_v_3.2 (1:100, NBP1-22444, Novus, Centennial, CO, USA) and rabbit anti-CCR2 (1:100, ab216863, Abcam, Cambridge, UK) incubated at 4 °C for 24 h. Next, three PBS washes were performed on the DRG sections, followed by incubating in secondary antibodies, which include Alexa Fluor 488 (donkey anti-mouse IgG, 1:250, 34106ES60, Yeasen, Gaithersburg, MD, USA) and Alexa Fluor 594 (donkey anti-rabbit IgG, 1:100, 34212ES60, Yeasen). The sections were placed in the darkroom for 2h, after which they were cleaned three more times with PBS and mounted with an anti-fluorescent attenuating sealer containing DAPI. All images were captured with a TCS SP8 confocal microscope (Leica, Germany) and further analyzed using Image J software (Fiji version, NIH). Three sections were randomly selected from each mouse for imaging and quantification.

### 2.8. CCR2 Antagonist and Agonist Treatment

To test the effect of CCR2 on somatic neurogenic inflammation and referred-mechanical hypersensitivity, mice were administered with an i.p. injection of 5 mg/kg CCR2 antagonist RS102895 (HY-18611, MedChemExpress, Monmouth Junction, NJ, USA) or the same volume of vehicle (10% DMSO) solution one week after operation. The detection of sensitized points and behavioral tests were performed 30 min after drug administration. Additionally, to substantiate the role of CCR2 in DRG during visceral pain, DRG samples were bathed with 100 nM of CCR2 agonist CCL2 (HY-P7764, MedChemExpress, Monmouth Junction, NJ, USA) or 10 μM RS102895 for T-type channel currents recording. All drugs were administered at the same doses as previously reported [23,24].

### 2.9. Statistical Analysis

Statistical comparisons of data obtained from the detection of sensitized points, behavioral tests, and immunofluorescence labeling in control and GU mice were conducted using the unpaired *t*-test. A two-way analysis of variance (ANOVA) with the post hoc test was employed to discern variations in current density and the Boltzmann fit curves between different groups. Differences in parameters of the Boltzmann fit formula among groups were evaluated using an unpaired *t*-test. Data are presented with significance set at *p* < 0.05.

## 3. Results

### 3.1. Neurogenic Inflammation and Referred Mechanical Hypersensitivity in Somatic Regions Induced by Gastric Ulcer

The H&E staining of gastric tissues in the control group displayed normal epithelial cells and no noticeable congestion or edema in the gastric mucosa (Figure 1B). In contrast, exposure to acetic acid resulted in significant gastric tissue damage, manifesting as the loss of epithelial cells, submucosal inflammatory cell infiltration, and reduced mucosal glands (Figure 1B). These results indicate that the injection of 20% acetic acid, but not of saline, is sufficient to induce gastric damage, implying that the mouse model of gastritis has been successfully established.

Plasma extravasation is widely acknowledged as a pivotal indicator of neurogenic inflammation. Evan’s blue, which exhibits a strong affinity for serum albumin, has been utilized to quantify the extent of extravasation accompanying inflammation [25]. To investigate whether visceral pain, such as that induced by GU, can be reflected on the body surface, causing a neurogenic inflammatory response, EB was administered via injection into the lateral caudal vein after GU modeling to quantify the extent of plasma extravasation. As shown in Figure 1C–J, the results suggested that the points of EB extravasation were predominantly distributed in the upper-back regions spanning the T9 to T11 segments, and at the operative incision in the GU mice, rather than in the control mice.

Moreover, to determine the referred mechanical hypersensitivity responses triggered by neurogenic inflammation in GU mice, von Frey filaments were employed to evaluate mechanical thresholds in the upper-back exudation points across the T9 to T11 segments. As illustrated in Figure 1K, the mechanical thresholds in the T9–T11 upper back exduation points of GU mice were lower than those in the control group (*p* < 0.0001), suggesting that the gastric ulcer induces somatosensory nociceptive sensitization in the upper back exudation points from the T9–T11 segments. Therefore, the upper back cutaneous exduation points from the T9–T11 segments are recognized as the sensitized points.

### 3.2. Acetic Acid-Induced Inflammation Increases the T-Type Ca^2+^ Currents (I_T-Type_) in DRG Neurons with Small Diameters but Not Medium Diameters

Based on the classical theory of DRR and AR, visceral inflammatory pain can affect cutaneous hypersensitivity through neurogenic inflammation, which is triggered by abnormal discharges of nociceptive neurons in DRGs that innervate the visceral organs [26]. Voltage-gated Ca^2+^ channels are critical for the active electrical properties of neurons, which are essential for propagating and modulating pain signals. In mice, DRG neurons are categorized as large- (>30 μm), medium- (20–30 μm), or small- (<20 μm) diameter [6]. DRG neurons with somatic diameters less than 30 μm are considered nociceptors as they can detect noxious stimuli and initiate pain responses [27]. To evaluate the differences in total calcium currents in T9–T11 small- and medium-diameter DRG neurons between control and GU mice, we employed a combination of voltage gating and pharmacological recording methods (Figure S1A). The membrane capacitances of different diameters DRG neurons are presented in Supplementary Table S1. As illustrated in Figure S1B, the *I*_Ca_ current densities (*p* < 0.05) and peak amplitude of *I*_Ca_ current (*p* < 0.01) in small-diameter DRG neurons from the GU group were markedly greater than those observed in the control group. Conversely, there are no significant differences in *I*_Ca_ current densities and peak amplitude of *I*_Ca_ current in the medium-diameter DRG neurons between the two groups (Figure S1D,E). These findings indicate that only the *I*_Ca_ currents of small-diameter DRG neurons exhibit significant changes in the GU group, potentially contributing to the action potential (AP) initiation and propagation, thereby further altering the neuronal excitability.

**Figure 2:**
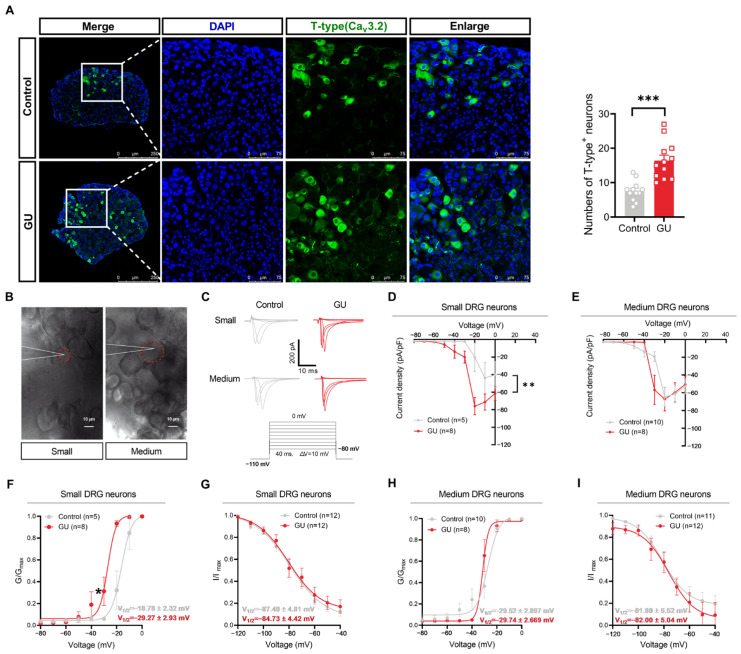
Both the expression and gating properties of the T-type channel in small-diameter DRG neurons are increased following acetic acid-induced GU. (**A**) Representative IHC images of DAPI (blue) and T-type Ca^2+^ channels (Ca_v_3.2) (green) in DRGs of control and GU groups. The right panels provide higher-magnification pictures of the boxed regions in the left panels [left: 10× (magnified), scale bar, 250 μm; right: 40× (magnified), scale bar, 75 μm]. The numbers of Ca_v_3.2-positive DRG neurons are shown in two groups (n = 12 DRGs from eight mice per group). Blue represents the cell nucleus, and green represents the T-type (Ca_v_3.2)-positive neurons. (**B**) Representative 40× (magnified) images of recording from a small-diameter (<20 μm) and medium-diameter (20–30  μm)  DRG neurons in a whole-mount DRG preparation are recorded [scale bar, 10 μm]. The dashed red circles represent the morphology of the clamped cells. (**C**) Top: Representative traces of T-type channel activation curves from different diameter DRG neurons in two groups. Bottom: The voltage protocol used to activate the T-type channels. (**D**,**E**) An overview of the normalized (pA/pF) *I_T-type_* density versus voltage relationship from DRG small-diameter (**D**) and medium-dimeter (**E**) neurons [two-way RM ANOVA with multiple comparisons tests: small-diameter DRG neurons, F _(1, 11)_ = 11.53, *p* = 0.0060; medium-diameter DRG neurons, F _(1, 16)_ = 0.1683, *p* = 0.6870]. (**F**–**I**) Boltzmann fits for normalized conductance, G/G_max_, voltage relations for voltage-dependent activation (**F**,**H**), and inactivation (**G**,**I**) of small- and medium-diameter DRG neurons in two groups [two-way RM ANOVA with multiple comparisons tests: small-diameter DRG neurons, activation curves: F _(1, 11)_ = 6.172, *p* = 0.0303; inactivation curves: F _(1, 22)_ = 0.2834, *p* = 0.5998; medium-diameter DRG neurons, activation curves: F _(1, 16)_ = 0.01542, *p* = 0.9027; inactivation curves: F _(1, 21)_ = 1.001, *p* = 0.3284]. (Small-diameter DRG neurons: control, n = 5–12 cells from five mice, GU, n = 8–12 from five mice; medium-diameter DRG neurons: control, n = 10–12 cells from five mice, GU, n = 8–12 from four mice.). Compared with the control group, * *p* < 0.05, ** *p* < 0.01, *** *p* < 0.001.

T-type Ca^2+^ channels have the distinctive capacity to become activated after a minor cellular membrane depolarization. This characteristic enables T-type channels to function close to the resting membrane potential, thereby influencing neuronal excitability [12]. Then, to ascertain whether the increased total *I*_Ca_ current density observed in the small-diameter DRG neurons is predominantly mediated by T-type Ca^2+^ channels, we first evaluated the changes in the T-type channel expression of T9–T11 DRGs. The immunofluorescent staining revealed a significant enhancement in the number of Ca_v_3.2-positive DRG neurons in GU mice compared to control mice (Figure 2A, *p* < 0.001).

In addition to observing the expression of the T-type channel changes, we further isolated and recorded *I*_T-type_ (Figure 2B,C). The membrane capacitances of different diameters of DRG neurons are presented in Table 1. As shown in Figure 2D, patch-clamp recordings revealed significantly higher *I*_T-type_ densities in small-diameter DRG neurons of GU mice than those in the control group (*p* < 0.01). In these small-diameter DRG neurons of the control group, the *V*_1/2_ of T-type channel current was −18.78 ± 2.32 mV and the *k* was 2.33 ± 1.01, respectively, while in the GU group, the *V*_1/2_ was −29.27 ± 2.93 mV (*t* = 2.323, *p* = 0.0426, unpaired *t*-test) and the *k* was 1.43 ± 0.27 (*t* = 1.008, *p* = 0.3352, unpaired *t*-test). The activation curve of T-type current fitted by a Boltzmann function exhibited a 10.49 mV hyperpolarizing shift in the small-diameter DRG neurons of GU mice (Figure 2F, *p* < 0.05). Subsequently, the steady-state inactivation curve showed no significant differences in the small-diameter DRG neurons between control (*V*_1/2_ = −87.49 ± 4.81 mV, *k* = −14.42 ± 2.61) and GU (*V*_1/2_ = −84.73 ± 4.42 mV, *t* = 0.4220, *p* = 0.6771, unpaired *t*-test; *k* = −13.25 ± 3.54, *t* = 0.2664, *p* = 0.7924, unpaired *t*-test) groups (Figure 2G). Moreover, there was no obvious change in the *I_T-type_* densities of the medium-diameter DRG neurons between the two groups (Figure 2E). The *V*_1/2_ and *k* of the T-type activation curves in the medium-diameter DRG neurons between control (*V*_1/2_ = −29.52 ± 2.897 mV, *k* = 3.50 ± 1.05) and GU (*V*_1/2_ = −29.74 ± 2.669 mV, *t* = 0.047, *p* = 0.9629, unpaired *t*-test; *k* = 1.03 ± 0.32, *t* = 1.906, *p* = 0.0759, unpaired *t*-test) mice showed no obvious differences (Figure 2H). The steady-state inactivation curve showed no obvious differences in the medium-diameter DRG neurons between control (V_1/2_ = −81.80 ± 5.52 mV, *k* = −14.54 ± 4.26) and GU (V_1/2_ = −82.00 ± 5.04 mV, *t* = 0.0274, *p* = 0.9784, unpaired *t*-test; *k* = −5.83 ± 0.97, *t* = 2.075, *p* = 0.0505, unpaired *t*-test) groups (Figure 2I). These results suggest that both the gating properties and expression of T-type channel changes may contribute to the alterations of total *I_Ca_* current.

### 3.3. Inhibition of the Chemokine CCR2 Reduces I_T-Type_ in Small-Diameter DRG Neurons and Attenuates Somatic Neurogenic Inflammation and Referred Mechanical Hypersensitivity in Gastric Ulcer Mice

**Figure 3:**
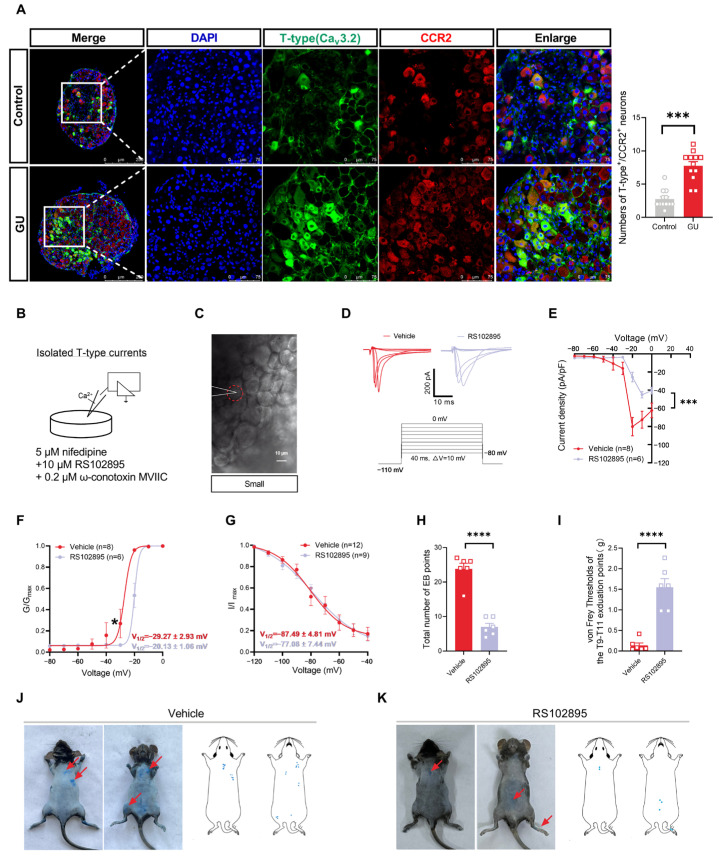
Effects of inhibition of the chemokine CCR2 on *I_T-type_* and referred somatic hyperalgesia in gastric ulcer mice. (**A**) Representative IHC images of DAPI (blue), T-type Ca^2+^ channels (green), and CCR2 (red) in DRGs of control and GU model. The right panels provide higher-magnification pictures of the boxed regions in the left panels [left: 10× (magnified), scale bar, 250 μm; right: 40× (magnified), scale bar, 75 μm]. The numbers of T-type (Ca_v_3.2)^+^ and CCR2^+^ DRG neurons are shown in two groups (n = 12 DRGs from eight mice per group). Blue represents the cell nucleus, green represents the T-type (Ca_v_3.2)-positive neurons and red represents the CCR2-positive neurons. (**B**) The schematic shows drugs and CCR2 antagonists added to the bath. (**C**) Representative 40× (magnified) image of recording from a small-diameter (<20  μm) DRG neurons in a whole-mount DRG preparation is recorded [scale bar, 10 μm]. The dashed red circles represent the morphology of the clamped cell. (**D**) Top: Representative traces of T-type channel activation curves from small-diameter DRG neurons treated with vehicle or RS102895 in GU mice. Bottom: The voltage protocol is used to activate the T-type channels. (**E**) An overview of the normalized (pA/pF) *I_T-type_* density versus voltage relationship from DRG small-diameter neurons [two-way RM ANOVA with multiple comparisons tests: *F* _(1, 13)_ = 8.014, *p* = 0.0142]. (**F**) Boltzmann fits for normalized conductance (G/G_max_) and voltage-dependent activation. (**G**) Boltzmann fits for voltage-dependent inactivation [two-way RM ANOVA withmultiple comparisons tests: activation curves, *F* _(1, 13)_ = 3.154, *p* = 0.1011; inactivation curves, *F* _(1, 22)_ = 0.006003, *p* = 0.9391] (vehicle, n = 8–12 cells from five mice; RS102895, n = 6–9 cells from four mice). (**H**) Quantification of total EB points in each group. (**I**) Withdrawal threshold to mechanical stimulation in the T9–T11 upper-back exudation points of each group (n = six mice per group). (**J**,**K**) Representative images on the left showing EB plasma extravasation points after i.p. of vehicle (**J**) or i.p. of RS102895; (**K**) 100 μL. Schematic representations of EB sites on the body surface of i.p. of vehicle (**J**) or i.p. of RS102895 (**K**) are on the right (n = six mice per group). The red arrows indicate the locations of EB points, and the blue dots represent the EB points. Compared with the vehicle group, * *p* < 0.05, *** *p* < 0.001, **** *p* < 0.0001.

CCR2 is the main receptor for CCL2 released from DRG neurons [28] and directly excites these nociceptive neurons by influencing their ion channels [6]. Previous research has shown that the upregulation of CCR2 on DRG is vital for pain induction, while CCR2 antagonists can induce analgesia [14]. Therefore, to clarify whether the alteration of T-type Ca^2+^ channel currents in the GU mice was mediated by the activation of CCR2 on T9–T11 DRG, we first evaluated the co-expression of CCR2 with Ca_v_3.2 within the T9–T11 DRG neurons in two groups. Immunofluorescent staining revealed a significant enhancement in co-labeled CCR2 and Ca_v_3.2-positive DRG neurons in the GU mice compared to the control group (Figure 3A, *p* < 0.001). Except for observing changes in CCR2 expression, we further investigated the role of CCR2 in modulating T-type Ca^2+^ channel currents by applying the CCR2 antagonist (RS102895,10 μM) to small-diameter DRG neurons in the GU group. The membrane capacitances of these neurons are presented in Table 1. The results indicated that the current densities of T-type Ca^2+^ channels in small-diameter DRG neurons of the RS102895 group were markedly decreased in comparison to those in the vehicle group (Figure 3E, *p* < 0.001). In the small-diameter DRG neurons of the vehicle group, the *V*_1/2_ and *k* were −29.27 ± 2.93 mV and 1.43 ± 0.27, respectively, while after washing the RS102895, the values became to −20.13 ± 1.06 mV (*t* = 2.588, *p* = 0.0237, unpaired *t*-test) and 1.30 ± 0.24 (*t* = 0.3380, *p* = 0.7404, unpaired *t*-test), respectively. The activation curve of T-type Ca^2+^ channel currents fitted by a Boltzmann function exhibited a notable 9.14 mV depolarizing shift in the small-diameter DRG neurons after treatment with RS102895 (Figure 3F, *p* < 0.05). Moreover, as illustrated in Figure 3G, the steady-state inactivation curve showed no significant difference in the small-diameter DRG neurons between the vehicle and RS102895 groups. These findings imply that the augmented T-type *I_Ca_* in small-diameter DRG neurons in GU mice may be mediated by the activation of CCR2 and that its antagonists can effectively reduce this activation.

Then, we investigated the effect of RS102895 (5 mg/kg), the CCR2 antagonist, on the number of EB exudate points and nociceptive responses in the GU mice. As illustrated in Figure 3, RS102895 markedly reduced the number of EB exudate points (Figure 3H, *p* < 0.0001) and enhanced the mechanical pain threshold (Figure 3I, *p* < 0.0001), thereby alleviating referred mechanical hypersensitivity in the somatic areas, particularly in the upper-back regions from T9 to T11 segments in GU mice compared to the vehicle group. These findings demonstrate that CCR2 is essential in the somatosensory nociceptive sensitization induced by viscera pain, potentially by mediating the gating properties of T-type channels in the small-diameter DRG neurons.

The above results highlight the essential relationship between neurogenic inflammation and referred mechanical hypersensitivity in somatic regions and the upregulation of CCR2 expression induced by visceral pain. Nevertheless, it remains unclear if activating CCR2 in the small-diameter DRG neurons of control mice can partially mimic the alterations in the gating properties of T-type channels associated with visceral pain. To investigate this, we assessed the *I_T-type_* in T9–T11 small-diameter DRG neurons of control mice treated with CCR2 agonist CCL2 (100 nM) during patch-clamp recordings. The membrane capacitances of these neurons are presented in Table 1. As illustrated in Figure S2, the T-type current density significantly increased with a hyperpolarizing activation curve shift underlying the role of exogenous CCL2 (*p* < 0.01). In the small-diameter DRG neurons of the vehicle group, the values of *V*_1/2_ and *k* of the T-type channel currents were −18.78 ± 2.32 mV and 2.33 ± 1.01, respectively, whereas after washing the CCL2, the values shifted to −27.50 ± 1.85 mV (*t* = 2.948, *p* = 0.0185, unpaired *t*-test) and 1.99 ± 0.52 (*t* = 0.3234, *p* = 0.7524, unpaired *t*-test). The activation curve of T-type Ca^2+^ channel currents, fitted by a Boltzmann function, exhibited a hyperpolarizing shift of approximately 8.72 mV in the small-diameter DRG neurons (Figure S2E, *p* < 0.05). These results suggest that exogenous CCL2 interacts with CCR2 in small-diameter DRG neurons of naive mice, potentially mimicking the effects of endogenous CCL2 acting on CCR2 in small-diameter DRG neurons of GU mice. The underlying mechanisms may involve the activation and upregulation of CCR2, which alters the dynamics of T-type channels.

## 4. Discussion

Although ion channels and chemokines in DRG neurons are known to participate in the pathogenesis of visceral pain, the distinct changes and regulatory mechanisms of T-type Ca^2+^ channel and CCR2 in different-sized DRG neurons after visceral injury are not well understood. Our study explored the underlying ion channel mechanisms of CCR2 on DRG neurons using acetic acid to construct a visceral pain model. We found that CCL2 directly increases T-type Ca^2+^ currents in normal small-diameter DRG neurons, which mimics the effects of endogenous CCL2 acting on CCR2, inducing the enhancement of T-type channel currents in GU mice. On the contrary, the administration of RS102895, a blocker of CCR2, reduced acetic acid-mediated referred somatic sensitization and T-type channel currents in small-diameter DRG neurons, which were distributed on the sensitized somatic area. These findings collectively indicated that the upregulation of CCR2 and T-type channel expressions, enhancing T-type channel currents in small-diameter DRG neurons, contributed to visceral pain-induced referred somatic hyperalgesia.

Visceral lesions may manifest as neurogenic inflammatory responses on specific somatic regions. Numerous studies have demonstrated that multiple EB extravasation points appear on the skin overlying the dorsal T6–T11 spinal cord segments in rat models of gastric injury, including minor curvature ulcers, acute and chronic gastric ulceration, and gastritis [29,30,31,32]. The quantity and area of EB extravasation points increase proportionally with the severity of gastric injury in mice [33]. These observations suggest that EB extravasation points are indicators of visceral pain-induced neurogenic inflammation, reflecting the cutaneous areas implicated in visceral pain disorders. In this study, using EB and von Frey tests, we noticed that in mice treated with acetic acid, the gastric referred area was primarily found on the upper back, innervated by T9–T11 segments, and within the gastric innervation. This neurogenic inflammation of GU mice is primarily driven by alterations in the functional activity of primary sensory neurons, particularly DRG neurons, leading to a lowered activation threshold and the heightened excitability of peripheral nerve endings that project to the affected areas, thereby resulting in the sensitization of referred somatic areas.

Referred somatic hyperalgesia is linked to the hyperexcitability of DRG neurons [34]. When the nervous system detects changes in the body’s internal environment, the membrane potential of sensory neurons may attain the threshold potential level, eliciting the generation of action potentials that encode corresponding sensory information in strings. Therefore, the alterations in the neuronal excitatory state constitute a critical prerequisite for sensory information transmission. Several studies highlighted the significant role of T-type Ca^2+^ channels in elevating the nociceptive neuron excitability by lowering the AP firing threshold and enhancing Ca^2+^ entry during AP propagation [35]. In this study, we concentrated on the small-diameter DRG neurons since they are identified as nociceptive neurons [6]. The results displayed that the mean values of T-type current density are 20.29 ± 16.20 pA/pF in the control and 75.96 ± 9.803 pA/pF in the model group at −20 mV, respectively, with CsCl internal solution. These findings diverge from those of other laboratories. For instance, a study, using the KCl internal solution, reported the T-type current density values of the small-sized (<25 pF) IB4-negative DRG neurons below 40 pA/PF at −20 mV in both control and model rats [36]. However, another one demonstrated the T-type current density values of the small-sized (<30 μm diameter) IB4-positive DRG neurons in model rats were markedly higher (76.66 ± 5.62 pA/pF) than control rats (43.33 ± 8.19 pA/pF) at −30 mV, utilizing a tetramethylammonium hydroxide internal solution [37]. Another study in rats, utilizing a Cs-methanesulfonate internal solution, recorded average peak T-type currents density values of 25 ± 2 pA/pF in normal and 93 ± 8 pA/pF in T-rich IB4-positive small-diameter (26–31 μm) DRG neurons at −20 mV [38]. Compared with these studies, such discrepancies may be attributed to a variety of factors, including the different DRG segments, electrophysiological recording conditions (e.g., temperatures, internal solutions, and voltage protocols), cell types (peptidergic or non-peptidergic), and species-specific differences in Ca_v_3 (T-type) isoform expression between mice and rats, as well as age-related differences influencing neuronal activity and T-type current densities. In brief, our findings revealed notable increases in both the current density and expression of T-type Ca^2+^ channels in small-diameter DRG neurons, whereas no such changes were detected in medium-diameter DRG neurons in GU mice. These enhancements may contribute to the hyperexcitability of small-diameter DRG neurons following referred visceral pain, thereby promoting the releases of the neuropeptides such as calcitonin-gene-related peptide (CGRP), histamine (HA), and substance P to referred sensitized areas, which can induce the vasodilation and extravasation of plasma proteins, resulting in referred somatic pain [39]. The functional alterations of T-type channel current in small DRG neurons may modulate their neuronal excitabilities and AP generations which is paramount to comprehending their pathophysiological significance in referred somatic pain models. Hence, future investigations should explore the AP threshold (rheobase), firing frequency, and additional membrane properties, which collectively reflect the intrinsic excitability of small-diameter DRG neurons.

CCR2 is the principal receptor for CCL2, critical for recruiting pro-inflammatory monocytes to inflamed tissue [40]. The release of CCL2 can enhance the excitability of DRG neurons, thereby facilitating pain sensitization [41,42]. In alignment with the proalgesic action of CCL2, the knockout of CCR2 has been shown to alleviate inflammatory and neuropathic pain resulting from chronic injury [16,17,18]. Additionally, both the short-term blockade and depletion of Ca_v_3.2 have also demonstrated analgesic effects. Moreover, T-type Ca^2+^ channels can be inhibited by CCR2 antagonists [43]. Therefore, it is plausible that a combined Ca_v_3.2 and CCR2 antagonist may exhibit synergistic effects in pain treatment. Consistent with these findings, our data showed a large increase in DRG neurons co-labeled with CCR2 and Ca_v_3.2 in GU mice. The current densities of the T-type Ca^2+^ channels were significantly decreased, coinciding with a reduction in the number of EB extravasation points and the alleviation of referred mechanical hypersensitivity in GU mice following the administration of RS102895. Nevertheless, the potential possibility for the intraperitoneal injection of a CCR2 inhibitor to induce other adverse effects, alongside the necessity for long-term pharmacokinetic analysis in mice, requires further investigation. Meanwhile, in future studies, the application of various chemokine receptor antagonists to verify target specificity in referred hyperalgesia arising from visceral pain would be crucial in achieving a more profound and comprehensive understanding of this phenomenon. Additionally, exposure to CCL2 in normal small-diameter neurons increased the T-type Ca^2+^ channel currents to levels comparable to those observed in GU mice. Hence, the activation and upregulation of CCR2 appear to alter the gating properties of T-type Ca^2+^ channels, potentially serving as underlying mechanisms for referred somatic sensitization during the visceral pain states. Further investigation is warranted to determine whether the effects of CCL2 on DRG neurons with small diameters act directly on CCR2 receptors, subsequently influencing the gating properties of T-type Ca^2+^ channels. In our research, we exclusively utilized the patch-clamp method to confirm the regulatory effect of CCR2 on T-type Ca^2+^ channels. Further exploration utilizing a wider array of experimental methods is necessary to elucidate the specific mechanisms of interaction between CCR2 and T-type Ca^2+^ channels. Whether CCR2 antagonist can exert long-term effects is also a key focus of our future research. Additionally, it remains to be established whether the administration of CCL2 to naive mice can induce somatic hyperalgesia.

The limitations of our study include the following points: (1) We currently categorize nociceptive neurons within DRG cell populations based on soma size and average capacitance; however, these approaches prove insufficient for a comprehensive characterization. Given these limitations and inadequacies, future studies are necessary to ascertain whether these small-diameter DRG neurons involved in the referred pain exhibit peptidergic or non-peptidergic properties. Such studies should integrate molecular profiling (e.g., IB4, TRPV1, CGRP) to more precisely define neuronal subtypes. For instance, IB4-positive neurons typically correspond to non-peptidergic C-fibers, whereas TRPV1- or CGRP-positive populations are linked to peptidergic nociceptive pathways [36]. These markers will enhance cross-species comparisons, particularly addressing size discrepancies in homologous phenotypes (e.g., rat DRG neurons with analogous functions). (2) Considering in vitro experiments, such as the patch-clamp technique employed in our work, cannot directly elucidate the causal relationship between hyperalgesia and DRG neuronal activities nor the specific mechanisms underlying the interaction between CCR2 and T-type Ca^2+^ channels, it is essential that future studies incorporate more sophisticated *in vivo* methodologies, such as calcium imaging, single-cell sequencing, co-immunoprecipitation, and cryo-electron microscopy to enhance our understanding.

## 5. Conclusions

The present study demonstrates that the activation of T-type Ca^2+^ channels in small-diameter DRG neurons is associated with GU-related neurogenic plasma extravasation and referred somatic allodynia. CCR2 may act as a regulator of neurogenic inflammation and mechanical allodynia by modulating the expression and gating properties of the T-type Ca^2+^ channels. The inhibitor of CCR2 may be a useful and novel therapeutic tool for visceral pain treatment.

## Figures and Tables

**Table 1:** The capacitance of different diameters DRG neurons in recording T-type Ca^2+^ currents.

Type	Group	Cm (pF)
Small diameter	Control	23.25 ± 1.74
	GU	21.85 ± 2.05
	RS102895 (CCR2 antagonist)	23.46 ± 2.30
	CCL2 (CCR2 agonist)	21.24 ± 1.02
Medium diameter	Control	33.92 ± 1.76
	GU	34.51 ± 1.74

## Data Availability

The original contributions presented in this study are included in the article/Supplementary Materials. Further inquiries can be directed to the corresponding authors due to ethical (animal research) reasons.

## Supplementary Materials

The following supporting information can be downloaded at: https://www.mdpi.com/article/10.3390/brainsci15030255/s1. Figure S1: GU induces the enhancement of total Ca^2+^ currents (*I_Ca_*) in small-diameter DRG neurons; Figure S2: CCR2 agonist increases T-type Ca^2+^ currents in normal DRG small-diameter neurons; Table S1: The capacitance of different diameters DRG neurons in recording total Ca^2+^ currents.

## Abbreviations

The following abbreviations are used in this manuscript:

CCR2	The C-C chemokine receptor 2
GU	Gastric ulcer
DRG	Dorsal root ganglion
CNS	Central nervous system
DRR	Dorsal root reflex
AR	Axon reflex
PNS	Peripheral nervous system
VDCCs	Voltage-dependent Ca^2+^ channels
HVA	High-voltage-activated
LVA	Low-voltage-activated
IGF-1	Insulin-like growth factor 1
GPCRs	G protein-coupled receptors
CCL2	The C-C motif chemokine ligand 2
NIH	The National Institutes of Health
CON	Control
PFA	Paraformaldehyde
H&E	Hematoxylin and eosin
EB	Evan’s Blue
PBS	Phosphate-buffered saline
ACSF	Artificial cerebrospinal fluid
i.p.	Intraperitoneal
AP	Actin potential
CGRP	Calcitonin-gene-related peptide
HA	Histamine
