# Supplementary material for: CCR2 Regulates Referred Somatic Hyperalgesia by Mediating T-Type Ca2+ Channel Currents of Small-Diameter DRG Neurons in Gastric Ulcer Mice
Source: Brain Sci. 2025 Feb 27;15(3):255. doi: 10.3390/brainsci15030255 (PMC11940306; doi:10.3390/brainsci15030255)
Supplement: Supplementary file 1 [file brainsci-15-00255-s001.zip › brainsci-3457765-supplementary.pdf]

Supplementary Materials

Supplementary figures and legends

Figure S1

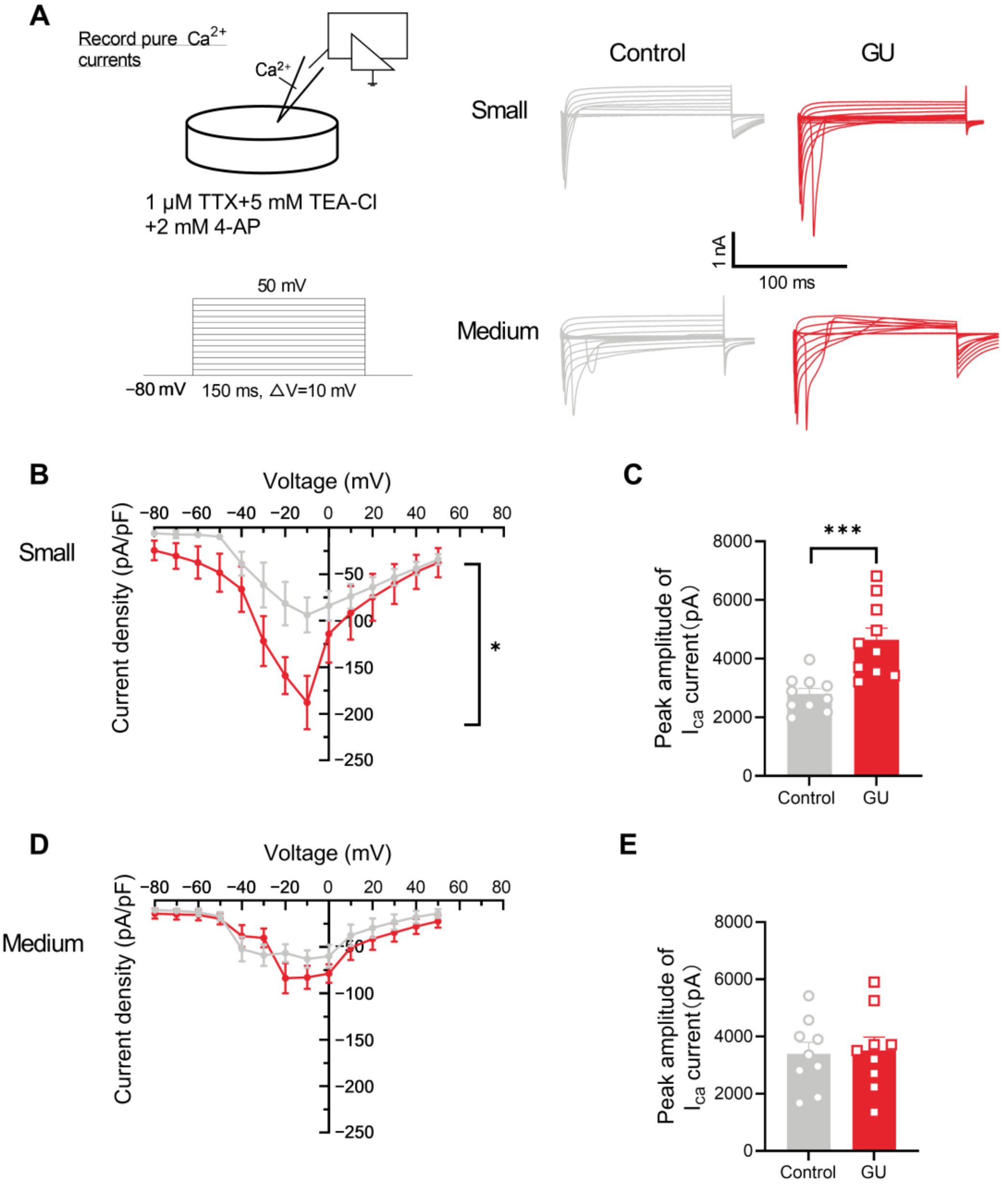

Figure S1. GU induces the enhancement of total Ca^{2+} currents (I_{Ca}) in small-diameter DRG neurons. **(A)** Left: The top schematic shows drugs applied to the bath. The down picture shows the protocol used to record the I_{Ca}. Right: Representative traces of I_{Ca} from small- and medium-diameter DRG neurons in the control and GU groups. **(B-E)** An overview of the normalized (pA/pF) I_{Ca} density versus voltage relationship and peak amplitude of I_{Ca} from the small-diameter **(B, C)** and medium-diameter **(D, E)** DRG neurons in two groups [B and D: Two-way RM ANOVA with multiple comparisons tests: small-

diameter neurons, $F_{(1, 20)} = 6.589$, $p = 0.0184$; medium-diameter DRG neurons, $F_{(1, 17)} = 1.052$, $p = 0.3194$; C: two-tailed unpaired *t*-tests: $t_{(df=18)} = 4.162$, $p = 0.0006$; D: two-tailed unpaired *t*-tests: $t_{(df=16)} = 0.1817$, $p = 0.8581$]. Control groups: small-diameter neurons, n = 10 from five mice, medium-diameter neurons, n = 10 from five mice; GU groups: small-diameter neurons, n = 9 from five mice, medium-diameter neurons, n = 9 from eight mice. Compared with the Control group, $^{*}p<0.05$, $^{***}p<0.001$.

Supplementary Table S1 The capacitance of different diameters DRG neurons in recording total Ca^{2+} currents

Type	Group	Cm (pF)
Small diameter	Control	19.97 ± 1.11
	GU	22.00 ± 1.18
Medium diameter	Control	38.69 ± 1.94
	GU	34.94 ± 1.11

Figure S2

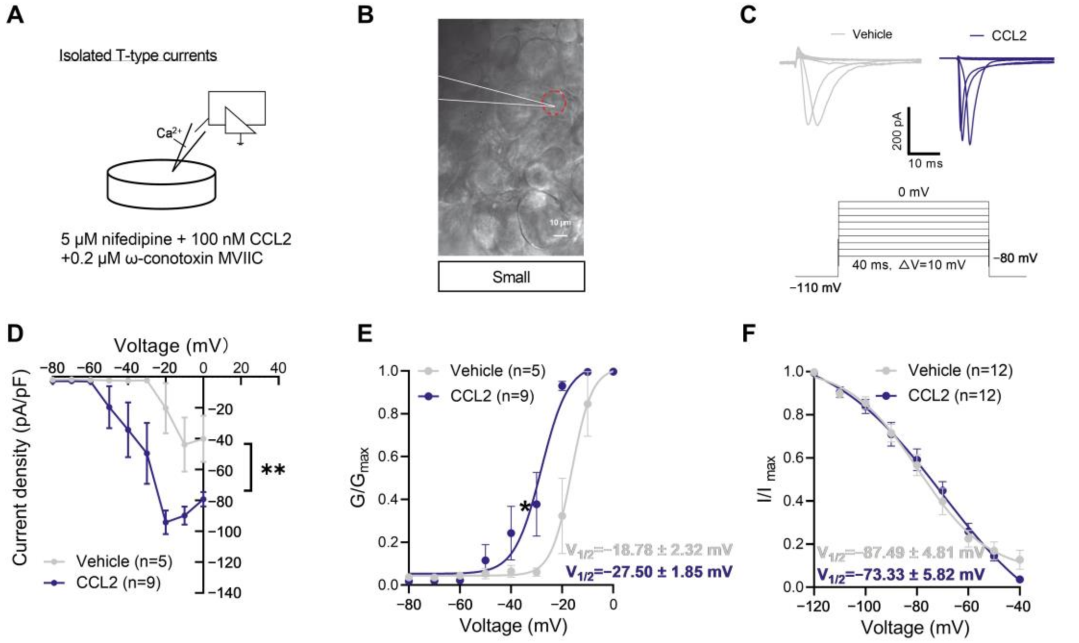

Figure S2. CCR2 agonist increases T-type Ca^{2+} currents in normal DRG small-diameter neurons. **(A)** The schematic shows drugs and CCR2 agonists applied to the bath. **(B)** Representative 40× (magnified) image of recording from a small-diameter (<20 μm) DRG neurons in a whole mount DRG preparation is recorded [scale bar, 10 μm]. The dashed red circles represent the morphology of the clamped cell. **(C)** Top: Representative traces of T-type channel activation curves from small-diameter DRG neurons treated with vehicle or CCL2 in wild-type mice. Down: The voltage protocol is used to activate the T-type channels. **(D)** An overview of the normalized (pA/pF) $I_{T\text{-}type}$ density versus voltage relationship from DRG small-diameter neurons [Two-way RM ANOVA with multiple comparisons tests: $F_{(1, 12)} = 9.358$, $p = 0.0099$]. **(E)** Boltzmann fits for normalized conductance (G/G_{max}) and voltage-dependent activation. **(F)** Boltzmann fits for voltage-dependent inactivation [Two-way RM ANOVA with multiple comparisons tests: activation curves, $F_{(1, 12)} = 7.653$, $p = 0.0171$; inactivation curves, $F_{(1, 27)} = 0.008760$, $p = 0.9263$]. (Vehicle, n = 5-12 cells from five mice, CCL2, n = 9-12 from three mice). Compared with the

Vehicle group, $^{**}p<0.01$.
